# Empirical Multiscale Networks of Cellular Regulation

**DOI:** 10.1371/journal.pcbi.0030207

**Published:** 2007-10-19

**Authors:** Benjamin de Bivort, Sui Huang, Yaneer Bar-Yam

**Affiliations:** 1 Department of Molecular and Cellular Biology, Harvard University, Cambridge, Massachusetts, United States of America; 2 Vascular Biology Program, Children's Hospital and Harvard Medical School, Boston, Massachusetts, United States of America; 3 Department of Pathology, Children's Hospital and Harvard Medical School, Boston, Massachusetts, United States of America; 4 Department of Surgery, Children's Hospital and Harvard Medical School, Boston, Massachusetts, United States of America; 5 New England Complex Systems Institute, Cambridge, Massachusetts, United States of America; Weizmann Institute of Science, Israel

## Abstract

Grouping genes by similarity of expression across multiple cellular conditions enables the identification of cellular modules. The known functions of genes enable the characterization of the aggregate biological functions of these modules. In this paper, we use a high-throughput approach to identify the effective mutual regulatory interactions between modules composed of mouse genes from the Alliance for Cell Signaling (AfCS) murine B-lymphocyte database which tracks the response of ∼15,000 genes following chemokine perturbation. This analysis reveals principles of cellular organization that we discuss along four conceptual axes. (1) Regulatory implications: the derived collection of influences between any two modules quantifies intuitive as well as unexpected regulatory interactions. (2) Behavior across scales: trends across global networks of varying resolution (composed of various numbers of modules) reveal principles of assembly of high-level behaviors from smaller components. (3) Temporal behavior: tracking the mutual module influences over different time intervals provides features of regulation dynamics such as duration, persistence, and periodicity. (4) Gene Ontology correspondence: the association of modules to known biological roles of individual genes describes the organization of functions within coexpressed modules of various sizes. We present key specific results in each of these four areas, as well as derive general principles of cellular organization. At the coarsest scale, the entire transcriptional network contains five divisions: two divisions devoted to ATP production/biosynthesis and DNA replication that activate all other divisions, an “extracellular interaction” division that represses all other divisions, and two divisions (proliferation/differentiation and membrane infrastructure) that activate and repress other divisions in specific ways consistent with cell cycle control.

## Author Summary

In a eukaryotic organism such as the mouse, the complete transcriptional network contains ∼15,000 genes and up to 225 million regulatory relationships between pairs of genes. Determining all of these relationships is currently intractable using traditional experimental techniques, and, thus, a comprehensive description of the entire mouse transcriptional network is elusive. Alternatively, one can apply the limited amount of experimental data to determine the entire transcriptional network at a less detailed, higher level. This is analogous to considering a map of the world resolved to the kilometer rather than to the millimeter. Here, we derive from mouse microarray data several high-scale transcriptional networks by determining the mutual effective regulatory influences of large modules of genes. In particular, global transcriptional networks containing 12 to 72 modules are derived, and analysis of these multiscale networks reveals properties of the transcriptional network that are universal at all scales (e.g., maintenance of homeostasis) and properties that vary as a function of scale (e.g., the fractions of module pairs that exert mutual regulation). In addition, we describe how cellular functions associated with large modules (those containing many genes) are composed of more specific functions associated with smaller modules.

## Introduction

**Figure 1:**
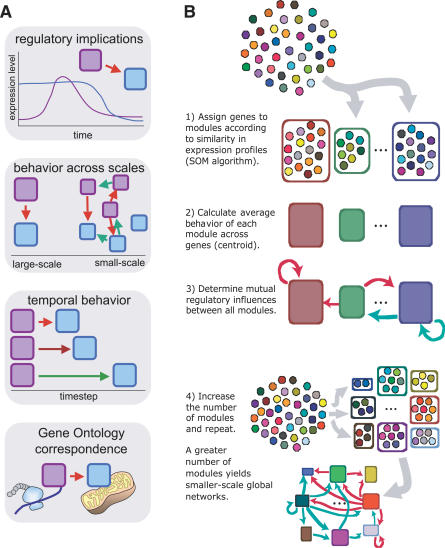
Schematics of Analytic Approaches and Network Determination Algorithm (A) Networks of module regulation were analyzed along four conceptual axes: (1) regulatory implications—if module 1 (purple) represses module 2 (blue), then an increase in the expression of module 1 will trigger a later reduction in the expression level of module 2; (2) behavior across scales—modules 1 and 2 are composed of genes that comprise parts of modules at finer scales with their own regulatory interactions; (3) the effect of module 1 on module 2 may vary depending on the length of the intervening time interval; and (4) module 1 and 2 may correspond to processes described by the GO database, such as protein synthesis or mitochondria. (B) Schematic of the algorithm used to generate multiscale networks of regulation from the same global collection of genes. Genes are divided into groups by similarity of behavior, and the mutual regulatory influences are determined. The process is repeated with a larger number of groups.

The importance of modular organization in biology is widely appreciated [1–6] and is manifested in conserved gene modules across species [7–9]. High-throughput data has yielded progress in molecular-level descriptions of interactions of genes, proteins, and metabolites [10–14]; however, understanding an entire cell or its major components from genetic information is a major methodological challenge [15]. Here, we use genome-wide expression Alliance for Cell Signaling (AfCS) data to first empirically obtain modular functions and then empirically obtain the effective inhibitory and activating regulatory influences between these modules at many scales of resolution (see Figure 1). This approach yields copious results about effective regulatory interactions so that a complete discussion is not possible in a single manuscript. Thus, we approached the results in the manner of other high-throughput and genome-wide analyses, presenting general principles that apply across all the data as well as a selection of individual observations that are discussed in greater detail in Text S1. The distinct approaches we use to analyze the results are diagrammed in Figure 1A.

The technique used to infer these regulatory influences relies on the correlation of the expression levels of transcriptional regulators at one time with the expression levels of their regulatory targets a fixed interval of time later. This correlative analysis aggregates direct and indirect causal influences, and co-occurring behaviors. Still, the transition matrix obtained can be used to predict [15] the transcriptional level changes of large cellular modules over fixed time intervals with surprising accuracy (*r* > 0.95). This approach generates many specific results, each of which is the strength and polarity (activating or inhibiting) effective regulatory influence of one functional module on another. The results are derived directly from experimental data and are statistically validated.

This multiscale analysis yields a description of cell behavior in terms of traditional biological concepts (i.e., cellular or physiological systems such as “respiration” and “mitosis”), identifying the genes whose collective behavior they comprise. At all scales, new network models of regulatory interactions among modules encompassing the behavior of the *entire* cell are presented. Previous studies have considered genome-wide multiscale groupings of genes according to their expression behavior [16–18]. This work extends the paradigm of multiscale gene grouping by determining for the first time, at multiple scales, the network of mutual regulation of groups of genes on groups of genes, an approach that is analogous to having geographic maps of varying resolution.

Our analysis of attributes of these novel cellular-level regulatory networks reveals principles of organization, such as scale-dependent homeostatic feedback and target specificity, and asymmetric restrictions on the number of ingoing and outgoing regulatory influences. Knowledge gained that is unique to multiscale analysis includes how functions of smaller modules contribute to the aggregate function of larger modules, which is analogous to how physiological systems are composed of organs, and organs out of tissues.

We provide all of the networks and Gene Ontology (GO) data in the supporting information, as well as a discussion of the statistical methods used to identify them. Specific questions about interactions between cellular modules can be addressed with these databases, as well as general insights or quantitative models of cellular response to perturbations. In Text S1, we discuss in detail samples of (1) randomly chosen and (2) particularly intriguing regulatory relationships inferred from our analysis. These examples can be considered at length, as they provide a large number of specific insights into the complex biological functioning of the cell, manifesting the ability of our methodology to extract them, and the informational value of the large AfCS datasets. Given the high complexity of cellular function, it should not be expected that a simple summary of modular interactions would serve as a sufficient description of the large number of results obtained. In the Results section, we focus on a selection of results that demonstrate the variety of interesting results that were found and general principles that have been abstracted from them.

## Results

### Regulatory Implications

**Figure 2:**
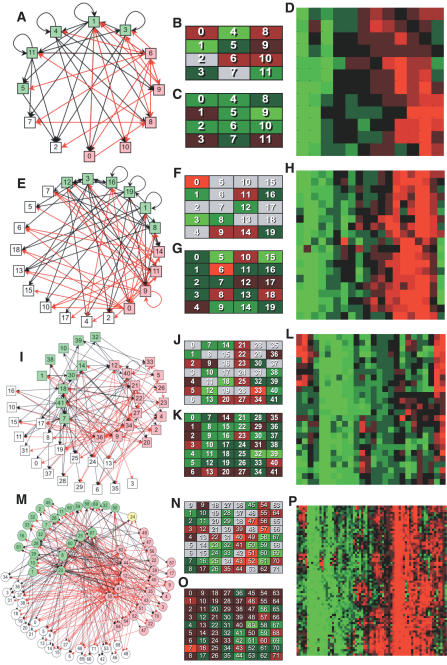
Effective Regulatory Influences at Many Scales (Top to Bottom) over a 1.5-h Interval (A,E,I,M) Regulatory influence networks, with red arrows indicating inhibition and black activation. An influence is included if the magnitude of its mean across all regulatory contexts is more than twice its standard deviation. Predominantly activating modules (green boxes), predominant inhibitors (red boxes), and modules without outputs (white boxes) are adjacent. Numbers in boxes are SOM module numbers. (B–C,F–G,J–K,N–O) Average magnitudes of outputs (B,F,J,N) and inputs (C,G,K,O) of modules arranged in SOM array order. Stronger inhibition, activation, or neither are indicated by brighter red, green, or gray, respectively. (D,H,L,P) Complete unthresholded *n* × *n* regulatory transition matrices. Rows are sorted by similarity in functional inputs, and columns are sorted by similarity in functional outputs.

The mutual regulatory influences for networks comprising *n* = 12, 20, 42, and 72 cellular modules are shown in Figure 2 for the 1.5-h time-interval (Table S3). These regulatory networks reveal a wealth of information about regulation at the cellular level. For the *n* = 12 scale, we have previously reported a number of key results [15]. Considering the new results at the *n* = 20 scale, module 3 is a global (pleiotropic) activator. Appropriately, module 3 contains a significant overabundance of genes involved in aerobic respiration (Table S4). Since these genes contribute to increased cellular energy, it is not surprising that module 3 is a ubiquitous activator of transcription. Module 9 is activated by module 3, and has a statistically significant association only with mitosis. Cell proliferation is known to suppress transcription [19], so it is appropriate that module 9 is a repressor of many modules, including itself. Its targets include module 3, one of many examples of negative/homeostatic feedback, consistent with the known coupling of respiration and the cell cycle [20]. Some modules have no regulatory outputs in these networks, e.g., module 15, which has an overabundance of genes involved in nucleosome assembly. This is surprising because increased DNA binding by nucleosomes correlates with lower transcriptional levels [21,22]. Examples of positive autoregulation can also be found; module 16 is associated with oxygen regulation and is self-activating. The specific strengths of these and all other effective influences are given in Table S3, and Text S1 provides a discussion of many additional specific regulatory interactions, including intuitive and surprising examples.

We calculated the average of the influence outputs (Figure 2B, 2F, 2J, and 2N) of each module on its targets, as well as the average input to each module (Figure 2C, 2G, 2K, and 2O), shown in Figure 2 in self-organizing map (SOM) array order (i.e., modules with similar expression are proximal). It is striking, particularly for *n* > 12, that while every module has several inputs, many modules have no outputs (shown in gray). These correspond to modules that are nonregulatory. Not surprisingly, similar expression responses (adjacency in the SOM arrays) more often correspond to input similarity than to output similarity.

To convey the distribution of all interactions, from strongly activating to strongly inhibiting, we cluster-ordered [23] the rows and columns of the *n* × *n* transition matrices at each scale (Figure 2, right panels). Rows were ordered by similarity in functional input and columns by similarity in functional output, since the value at row *i*, column *j* is the effect of module *j* on module *i*. Vertical bands in the matrices imply that modules tend to have a uniform output, a property absent in randomized controls (unpublished data). These global influences may reflect a restricted energy economy in which consumption or production transcriptionally inhibits or activates all other cellular activities [15].

A goal of developing larger-scale models is to relate genetic function to conceptually accessible models of cellular function. Still, even at the largest scale given above, with 12 modules and 144 potential interactions, it is hard to develop a complete mental picture of the behavior of the cell. We therefore developed an even more accessible, larger-scale summary of cellular function, which can serve as a first guide to the understanding of cell behavior at all finer levels of organization. Inspecting the regulatory effects of the groups reveals that the cell transcription network at *n* = 12 can be partitioned into five functional divisions: (1) energy and component production, (2) proliferation and differentiation, (3) extracellular interaction, (4) membrane infrastructure, and (5) DNA replication. Groups 0, 1, and 4 comprise the first division. They are all enriched for genes involved in ATP synthesis and the production of nucleic acids and proteins, and are appropriately all global activators of transcription over short time-scales (1.5 h). Each group has unique sub-behaviors, with group 0 involved in endocytosis and group 1 in apoptosis and protein folding. Group 4 is involved in cell-cycle regulation (group 4 is also a global activator over 1 h), providing a connection between energy production and proliferation.

Groups 2 and 3 belong to the division contributing to proliferation and differentiation. They are both enriched for genes involved in small-molecule metabolism, and unlike the previous division, they are not global activators of transcription. Instead, they activate their own division over all time-scales and inhibit the DNA replication division (below), particularly after 1 and 3 h. This periodic repression of DNA replication by the proliferation division may provide for the timing of S-phase during the cell cycle. Group 2 shares some functions with the first division, containing genes involved in translation and transcription, but has no role in ATP synthesis. Like group 4, it is involved in regulating the cell cycle. Group 3 has specific sub-functions related to the immune response, cell adhesion, and, more generally, behaviors unique to particular cell types.

Groups 5, 8, and 9 are unified by gene content related to interactions between the cell and its external environment (division 3). They are respectively enriched for genes involved in cell matrix/adhesion and endocytosis; oxygen transport and exocytosis; and oxygen transport, chemotaxis, and the immune response. Functionally, all of these genes are global repressors of transcription after 1.5 h, with regulatory influences that are in opposition to the energy and component production division. Group 5 is also a weak early (0.5–1 h) activator of global transcription.

Groups 6 and 7 comprise the membrane infrastructure division, with both enriched for genes involved in the physical production and maintenance of the cell membrane, such as lipid metabolism, lipid catabolism, and cholesterol metabolism. These groups are generally self-activating, but do not exert strong global transcriptional influences on other groups over any time interval, consistent with their infrastructural role. Unlike other divisions, these groups are virtually indistinguishable in terms of gene function.

Last, groups 10 and 11 form the DNA replication division. They are enriched for nucleotide synthesis, nucleosome assembly, and regulators of DNA methylation. They are both strong global activators of transcription 2 h after they are activated, and while group 11 is at other times a weak global activator of transcription, group 10 is essentially not globally regulatory over other intervals. Group 10 is also uniquely enriched for genes promoting and suppressing apoptosis, and, like groups 8 and 9, has role in oxygen regulation.

### Behavior across Scales

**Figure 3:**
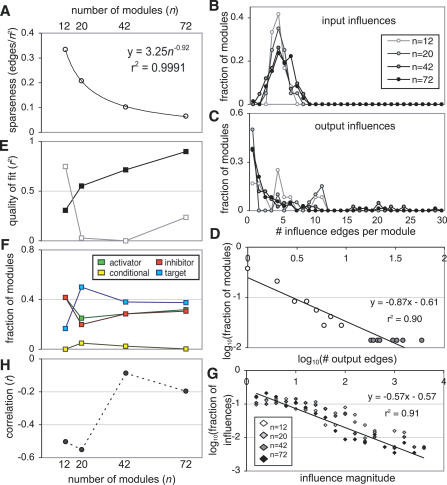
Scaling Properties of Regulatory Networks (A) Sparseness (number of regulatory interactions divided by the total number of possible interactions) plotted versus number of modules (scale). (B) Distribution of number of regulatory inputs at each scale. (C) Distribution of number of regulatory outputs at each scale. (D) Power-law fit of the module output number distribution in the *n* = 72 case. (E) The quality (*r^2^*) of power-law fits to module output (black) and input (gray) distributions versus scale, showing increasingly good fit for output distributions. (F) The fraction of modules that are activating (green), inhibiting (red), mixed activating and inhibiting (yellow), or nonregulatory targets (blue) versus scale. (G) Regulatory influence magnitude distributions at all scales (log-linear plot). The data are approximately exponential (line). Influences were included here if the standard deviation of their estimation replicates was less than 1, regardless of the mean. (H) The correlation (*r*) across modules of the average regulatory input and output versus scale. Note the negative *y*-axis scale.

We analyzed trends in the networks to determine which cellular properties hold across scales of observation and which vary with scale (Figure 3). The sparseness (identified interactions/total possible interactions; Figure 2, second column of panels) follows power-law scaling (Figure 3B). Specifically, while the number of possible edges grows as *n^2^*, the number of inferred influences grows linearly. This leads to progressively sparser and sparser matrices. For our four scales, this trend was very closely fit (*r^2^* = 0.9991) by a power-law with exponent −0.92. Extrapolating this trend gives very rough predictions of ∼39,250 regulating interactions in the 6,000-gene yeast genome and ∼197,000 in mammals. The former falls between the number expected for genetic interactions (97,000 [24] and 100,000 [25]) and the number of expected physical interactions (8,300 [26] and 6,300 [27]). Another study [28] gives an estimate of roughly 340,000 physical interactions in mammals.

At all scales the distribution of the number of inputs to each module is Gaussian (Figure 3B), while the number of outputs is better fit by a power-law (Figure 3C). This is particularly true as we consider finer scales (Figure 3D and 3E). We suspect this reflects an inherent limitation—while a module can broadcast regulation over a large number of target modules (i.e., the overrepresentation of high numbers of outputs in the power-law distribution), each module is limited in the number of inputs it can usefully accept. These properties are also seen in influence networks of engineering projects [29]. How this limitation is mechanistically imposed on a large module of genes is an interesting open question.

### Scaling of Target Specificity

At all scales, the number of modules with only activating outputs is nearly identical to the number of modules with only inhibiting outputs (Figure 3F). The magnitudes of influences appear exponentially distributed (Figure 3G) at all scales, implying a functional cutoff in influence strength. Given that the interaction of two modules is composed of the interactions of many pairs of constituent genes, this functional cutoff could be explained by assuming that individual gene interactions have a characteristic strength and occur effectively randomly. However, these assumptions may be too strong, and there may be selective advantages to bounding interaction strengths, such as limiting total module expression. We also considered the relationship between the average input and output of each module. For all *n*, these measures were anticorrelated (Figure 3H).

**Figure 4:**
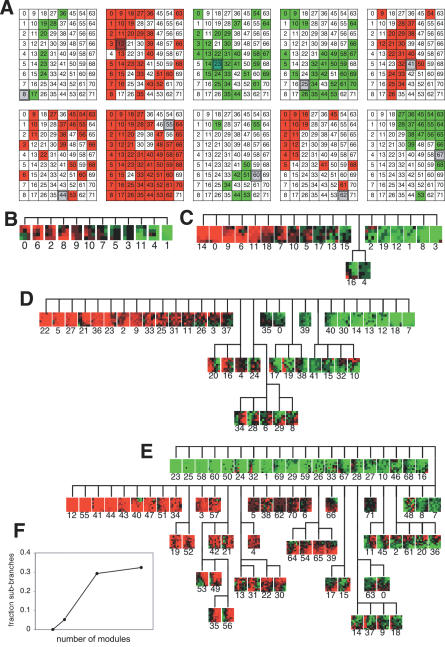
Targets of Module Regulation, Showing Increasing Specificity at Finer Scales (A) Examples of regulatory targets. Activated (green) or inhibited (red) modules of the regulatory module marked in gray. Influences passing the edge inclusion threshold are shown. Targeted modules tend to be spatially localized in the SOM array, i.e., modules expressed similarly across perturbations tend to receive inputs from the same activators and inhibitors). (B–E) Target influence SOM arrays (unthresholded) clustered by similarity at *n* = 12, 20, 42, and 72 (B–E, respectively). For example, the first array in (A) corresponds to module 8 in (E) (second row, second position from the right). (F) The degree of target specificity (fraction of branch points not on the longest arm of the clustering tree) versus network scale. The sigmoidal shape of this curve is suggestive of a transition around *n* = 30 from global regulation to higher target specificity.

At the finest scale of *n* = 72, the targets of a particular module output tended to have similar expression profiles. This was manifested in the frequent adjacency of modules' targets in the SOM array (Figure 4A). We would expect this result at finer scales, since modules with similar expression patterns should have similar regulators at fine scales. The modules were hierarchically clustered according to correlation in their effects on target modules using the Fitch-Margoliash algorithm (Figure 4B–4E). The coarser trees (*n* = 12 and 20) only show variation along a single dimension ranging from all-activating to all-inhibiting outputs, without sub-branches. This implies that outputs of each module tend to be uniform across all targets. More specific targeting at smaller scales appears as sub-trees branching off the linear portion of the tree (Figure 4D and 4E insets) composed of modules that activate some targets but inhibit others. This trend is quantified in Figure 4F, which shows the fraction of nodes in the tree that are on sub-branches. The shape of this curve suggests a transition from universal to target specificity at finer scales.

### Temporal Behavior

In addition to the 1.5-h influences, we determined the transition matrices for all other time intervals (0.5 h [i.e., the transition between 0.5 h and 1 h], 1 h, 2 h, 3 h, and 3.5 h) in the AfCS data at each scale (available in Table S5). We calculated the average output influence of each module as a function of time (Figure S1); modules with similar regulation under all assayed conditions (such as putative housekeeping modules and constitutively repressed modules) have lower-magnitude outputs than modules that are regulated in a situation-dependent manner. Modules with weak outputs tend to occur around the periphery of the SOM array, while modules with the greatest variance are in the interior. “Peripheral modules” tend to have monotonically increasing or decreasing responses to all perturbations, whereas the interior modules' responses are more complex.

Averaging the magnitudes of the mean outputs of each module over all the modules reveals which time intervals mediate the greatest changes in expression. At all scales the average influence magnitude varies periodically with time, with greater frequency at finer scales (i.e., for *n* = 12, the most potent influences occur over *t* = 1.5 h, and the least over *t* = 3 h, whereas for *n* = 42, those times are 1 h and 2 h, respectively; see Figure S1C and S1D). Moreover, the magnitude of influences decreases at finer scales. This supports the idea that smaller modules bring about smaller transcriptional changes over shorter times [6].

### Scaling of Gene-Module Function

**Figure 5:**
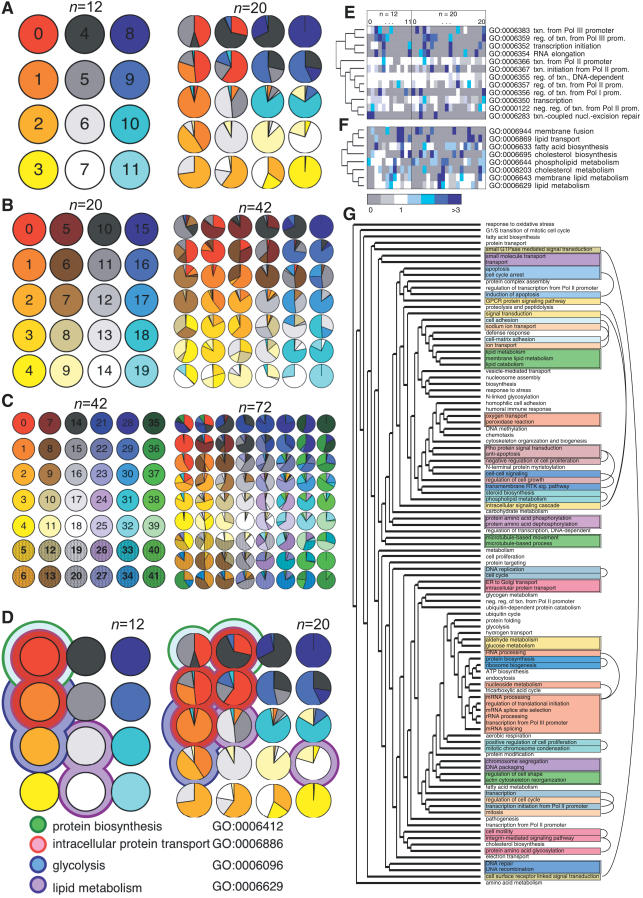
Contribution of Smaller-Scale Modules to Larger-Scale Modules and Aggregation of Ontological Function (A–C) Mappings of modules at finer scales (right array) to larger scales (left array). Size of a wedge of a particular color indicates the fraction of genes in that module that were placed in the larger-scale module of matching color. All arrays are in the SOM order. (D) Colored outer circles indicate representative GO category labels that are statistically overrepresented in modules at *n* = 12 and 20. Inner circles are the same as (A). (E) Individual GO labels within the GO category *transcription regulation* clustered by similarity of their enrichment fraction across the *n* = 12 and 20 SOM modules. Gray and blue indicate under- and overrepresentation, respectively. See scale. (F) Similar to (E) for the GO category *lipid processing.* (G) GO labels appearing 10 or more times clustered by distribution across the *n* = 12 and 20 modules. Doubly outlined boxes indicate clades with closely related function. Singly outlined boxes are within four branches of functionally related ontologies, indicated by the arcs.

We next considered the mapping of ontological terms for gene function between scales. To analyze how cellular functions are composed of sub-functions, we identified the mappings by which larger scale modules are composed of finer-scale modules. These mappings are shown in Figure 5A–5C, where the sources of modules at larger scales, in terms of the modules at the next finer scale, are shown by color-coding. The module partitioning is not strictly hierarchical, since the boundaries between modules at one scale need not align with the boundaries between modules at a different scale. Thus, a set of genes in a single module at smaller scales may be found across more than one module at larger scales. Moreover, the nonhierarchical organization of genes into behaviorally related modules is an observation derived from the data. If gene behavior were hierarchically organized, the SOM algorithm would have found fine-scale modules that were strict subsets of large-scale modules.

These mappings also show how ontological functions are distributed across modules at various levels as illustrated for four GO categories in Figure 5D. For example, in the *n* = 12 case, the “intracellular protein transport” function is coassociated with either ”protein biosynthesis” (in module 0) or “glycolysis” (in module 1), respectively, but at the *n* = 20 scale, “intracellular protein transport” occurs independently in modules 1 and 6, and in conjunction with “protein synthesis” in module 5 and “glycolysis” in module 2. In addition, the dissociation of the two modules labeled “intracellular protein transport” at *n* = 12 into four modules at *n* = 20 also illustrates how higher-specificity functions are aggregated to form larger-scale functions of lower specificity.

Finally, we examined to what extent the distribution of sub-functions across the multiscale SOM groupings can predict relatedness of GO function. Cluster analysis of a GO label's abundance similarity across the *n* = 12 and *n* = 20 SOM groupings (Figure 5E–5G) revealed that more closely related functions tend to have similar distributions, and are therefore proximal in the generated cluster tree; e.g., in Figure 5E, transcription elongation and initiation are adjacent in the tree, as are the Pol III–related functions. The deepest distinctions in the tree are based on differences in distribution at the *n* = 12 scale, while the shallowest branches are determined by the *n* = 20 scale. The clustering indicates that the hierarchy of GO functions (in an abstract functional sense) can be inferred solely from the distributions of genes across the SOM groupings. That the association between GO groups and multiscale SOM gene modules can be used to partially reconstruct the GO hierarchy implies that SOM module identity predicts a gene's function in terms of familiar large-scale cellular behaviors. This indicates that SOM grouping across levels provides an additional tool for identifying gene function.

## Discussion

We have determined networks of regulatory influences exerted by large groups of similarly behaving genes on other such large groups across multiple scales of resolution. These effective regulatory influences between modules are composed of direct and indirect causal mechanisms as well as temporally correlated effects that are seen across all 33 perturbations. Given a perturbation of gene expression, all of these components contribute to a prediction of the transcriptional state at later times [15]. Since the effective regulatory interactions accurately predict the transcriptional state, they capture almost all of the biologically relevant causal regulation occurring within that time interval.

We determined these effective regulatory interactions between gene modules at different scales of observations comprising between five and 72 components. The gene composition of these modules is not strictly hierarchical; i.e., two genes in the same fine-scale group may not belong to the same large-scale group. This is a natural consequence of imposing discrete classification categories onto systems that need not be hierarchically structured across scales. For example, if one were to classify visible colors into six categories, they might very well comprise red, orange, yellow, green, blue, and indigo. The hues “yellow-orange” and “yellow-green” might reasonably fall into the yellow category of this six-group partitioning. However, if one divides the same colors into three higher-scale categories—red, green, and blue—those two same hues would fall into separate categories (red and green, respectively). Similar considerations apply to physiological and metabolic categories. Therefore, our a priori expectation should be that gene modules would be nonhierarchical across multiple scales, as is observed in results of the SOM partitioning.

From these groups and regulatory influences, we derived many results comprising a first multiscale analysis of global gene-regulatory influences. We consistently observed mechanisms consistent with the maintenance of homeostatic equilibrium across the modules, particularly at higher scales. For example, the apparent regulatory dissimilarity of modules with similar expression patterns (Figure 2B, 2F, 2J, and 2N) likely reflects a homeostatic mechanism in which it is unfavorable to have all coactivated modules as either strong activators or strong inhibitors. This pattern is found at all scales analyzed. The near parity between the number of activating modules and inhibiting modules in networks of all analyzed scales (Figure 3F) could reflect a homeostatic requirement that the total rate of transcription remain roughly constant (provided regulatory strength and transcriptional rate are uncorrelated). We observe that the net input and average output of a module are negatively correlated, particularly at the highest scales. This can be understood as reflecting another homeostatic mechanism. If a module is on average an activator (inhibitor), its input tends to be inhibitory (activating) to avoid positive feedback that causes large fluctuations from equilibrium in the total transcriptional level. Interestingly, at finer scales, there is increasing correlation between average module input and output, reflecting the increasing possibility of positive feedback. Positive feedback often leads to bistability or multistability [30], a property required for developmental differentiation. Consistent with our finding of positive feedback at small scales, cell-fate decisions are typically controlled by small circuits of mutually regulatory “master genes” [31,32]. In contrast, homeostatic regulation (e.g., of basic metabolic states) involves large-scale biochemical networks where robustness to fluctuations is necessary for overall stability of cell function. Positive feedback would therefore be excluded at this highest scale to avoid unsustainable abrupt genome-wide changes in gene expression.

A multiscale approach is conceptually essential given the organization of living systems into structures at many scales, and is critical given the staggering challenge of obtaining a complete description of pairwise gene interactions. Still, in view of the complexity of biological function, there is a large amount of information that arises from a multiscale analysis. In this sense, our analysis can be considered as foundational to the development of many other results. It is a high-throughput analysis methodology analogous to high-throughput experimental methods of genome sequencing or gene expression data collection; through our approach, a seemingly overwhelming amount of data is generated by high-throughput consideration of the large number of regulatory interactions of modules across multiple scales. Our analysis of these results has been correspondingly multiscale. First, we identified global principles, such as the many facets of homeostasis and universality of regulatory effects at larger scales. Second, we found new patterns of multiscale organization, such as the dichotomous distributions of the number of regulatory inputs and outputs at various scales, the increased target specificity and speed of regulation at finer scales, and the aggregation of sub-module functions into collective larger-scale functions. Last, we provide detailed discussion of many specific regulatory relationships in Text S1. The diversity of analysis points the way to many new lines of investigation, in particular experimentally testable hypotheses at large scales of cellular organization.

## Materials and Methods

To separate genes into modules [1–9] and determine their mutual regulation, we used the AfCS murine B-lymphocyte perturbation expression data [33] tracking the response of ∼15,000 genes to 33 perturbations at four time points (0.5 h, 1 h, 2 h, and 4 h; see Figure 1B). These expression levels were aggregated to yield a ∼15,000 (genes) × 132 (conditions and times) dataset. Genes were categorized by similarity of expression changes across all perturbations into *n* different modules, where *n* was taken to have the values 12, 20, 42, and 72 (see Tables S1 and S2), using the SOM algorithm. This categorization occurs without any a priori assumptions about the distribution of the data, and thus the SOM groups convey the full diversity of expression profiles.

The SOM process organizes the modules into a 2-D array according to the relatedness of their average changes in expression [34] such that modules that are adjacent in the SOM array have more similar expression responses across all conditions. Generally, genes that had monotonic responses to many perturbations (i.e., always being activated or repressed) tended to be placed in the corner positions of this array. These groupings were performed using the GEDI software [35].

Varying *n* allowed us to consider global sets of modules at various scales of description. Low *n* yields large-scale modules with many genes in each module; higher *n* yields small-scale modules with fewer genes. A representative profile for each module was used to represent the modules' behaviors, and was determined as the centroid of the expression profiles of all genes composing the module. From the *n* by 132 = 33 × 4 “module transcriptional profile” datasets, we obtained the effective regulatory interactions as an *n* × *n* transition matrix (**M**), where **M** **×** **X** *_t_* = **X** *_t_*_+*k*_, and **X** *_i_* is the *n* × 1 transcriptional state at time *i*. If the matrices were dense, the greatest mathematically solvable *n* would be the number of perturbations, 33. However, the matrices are sparse, and we used a bootstrapping technique to obtain transition matrices as large as 72 × 72. This was done by randomly choosing 12 out of *n* modules, solving for their mutual interactions, and repeating this process until each of the *n* ^2^ interactions was estimated many times in different 12 × 12 sub-matrices. We constructed our regulatory networks out of only those interactions that were statistically reliable across perturbation and transcription contexts, using a signal to noise analysis (Protocol S1). The bootstrapping was performed using custom written C++ code, and the linear systems were solved using Mathematica (Wolfram Research, http://www.wolfram.com). Clustering trees were all generated using the Fitch-Margoliash method as implemented in the Phylip program [36].

## Supporting Information

Figure S1 Temporal Dependence of Regulation (A) The average output of each gene group (shown in SOM array order) as a function of the time-step for each gene group for the *n* = 20 scale. Time points range from 0.5 h to 3.5 h. The standard deviation across time steps is indicated by color (see legend).
(B) Similar to (A) for the *n* = 72 scale.
(C) The average magnitude of regulation across all gene groups versus time-step (blue). Gray curves show fit by sinusoidal waves.
(D) Frequency and magnitude of transcriptional regulation oscillations versus scale. The decrease in magnitude and increase in frequency indicates that regulation is weaker and quicker (higher frequency) at finer scales.
(152 KB PDF)

Protocol S1 Supporting Statistical Analysis (419 KB DOC)

Table S1 Gene Ontology Labels Associated with AfCS Probe IDs and Gene Indices (2.5 MB XLS)

Table S2 Assignment of Genes into SOM Groupings of Varying Size (1.5 MB XLS)

Table S3 Statistical Estimates of Interactions between Gene Groups (898 KB XLS)

Table S4 Statistical Association of Gene Ontology Labels with Gene Groups (466 KB XLS)

Table S5 Interactions between Gene Groups over Varying Time-Steps (1.1 MB XLS)

Text S1 Specific Regulatory Insights Gained from Analysis of Networks of Cellular Regulation (80 KB DOC)

## Abbreviations

AfCS: Alliance for Cell Signaling
GO: Gene Ontology
SOM: self-organizing map
