# Supplementary figures and images for: Empirical Multiscale Networks of Cellular Regulation
Source: PLoS Comput Biol. 2007 Oct 19;3(10):e207. doi: 10.1371/journal.pcbi.0030207 (PMC2041980; doi:10.1371/journal.pcbi.0030207)

Figure S1

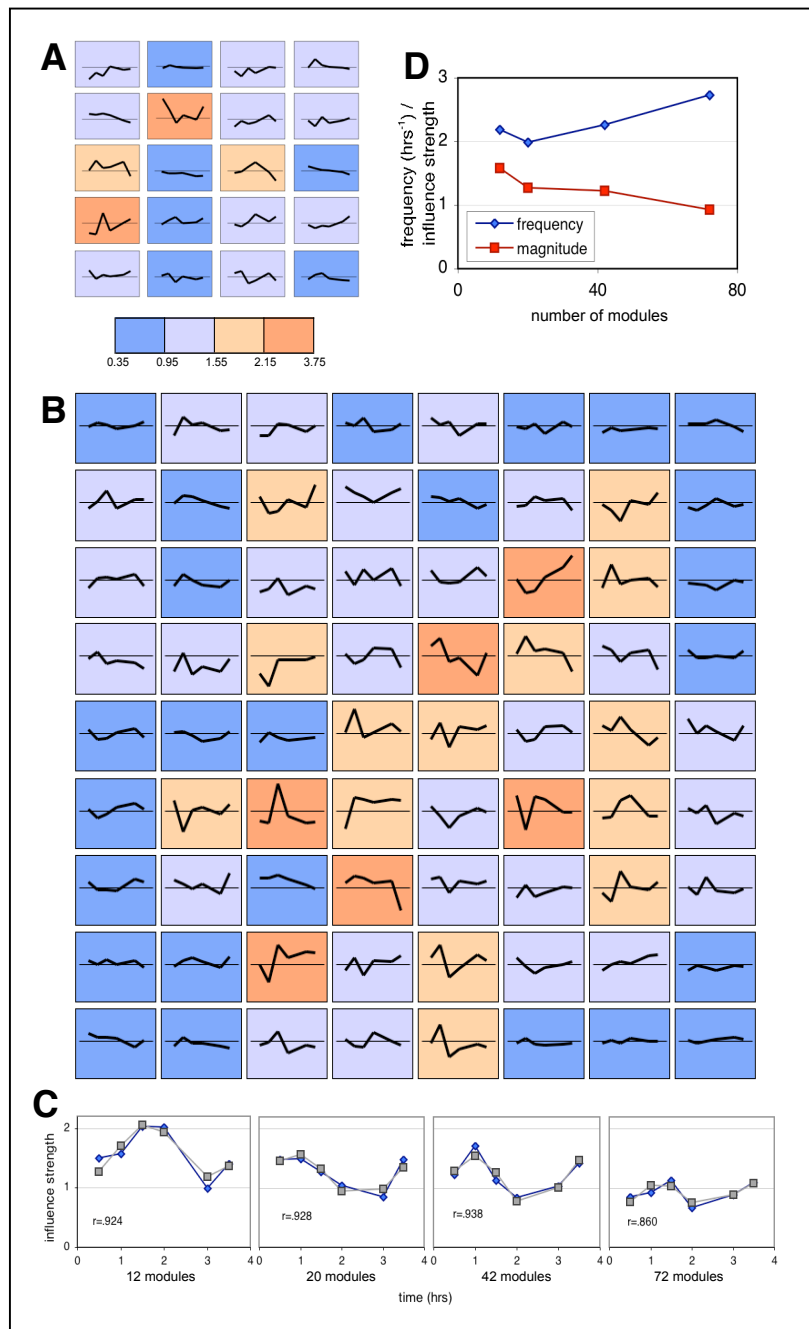

Supplement: Figure S1 — (A) The average output of each gene group (shown in SOM array order) as a function of the time-step for each gene group for the n = 20 scale. Time points range from 0.5 h to 3.5 h. The standard deviation across time steps is indicated by color (see legend). (B) Similar to (A) for the n = 72 scale. (C) The average magnitude of regulation across all gene groups versus time-step (blue). Gray curves show fit by sinusoidal waves. (D) Frequency and magnitude of transcriptional regulation oscillations versus scale. The decrease in magnitude and increase in frequency indicates that regulation is weaker and quicker (higher frequency) at finer scales. (152 KB PDF) [file pcbi.0030207.sg001.pdf]
