# Supplementary material for: Empirical Multiscale Networks of Cellular Regulation
Source: PLoS Comput Biol. 2007 Oct 19;3(10):e207. doi: 10.1371/journal.pcbi.0030207 (PMC2041980; doi:10.1371/journal.pcbi.0030207)
Supplement: Protocol S1 — (419 KB DOC) [file pcbi.0030207.sd001.doc]

**Protocol S1**

We obtained mutual regulatory networks of gene groups by statistically analyzing regulatory influences inferred from multiple experimental observations provided by AfCS. Details of the analysis are provided in this section of the supporting materials. The analysis we performed extracts the results and also evaluates their robustness to experimental noise, and shows that independent subsamples can be used to obtain the same results. The statistical approach included testing convergence of results using data subsampling, including only interactions that are robust across regulatory contexts, evaluating correlations between results obtained using different subsamples, and statistical tests of edge inclusion criteria.

An analysis of the convergence of the least-squares calculation of influences between gene groups with increasing data revealed that using 33 observations is sufficient to derive a transition matrix for the effective mutual regulatory influences of 12 gene groups. This is the case despite potentially high levels of noise in the microarray data. In particular, for the case of n=12 (see figure 1^1^). using only 26 observations yields transition matrices that are highly correlated (r>0.9) to the matrix derived with all the data. The same analysis can be used to obtain the mutual influences of any single set of 12 gene groups. For example, after identifying n=72 SOM gene groups, we can select 12 of them to obtain their mutual influences. The average correlation between derivations of the 144 mutual influences of gene groups 0 through 11 using 26, 28 and 30 random observations, and those using all 33 observations was 0.88, 0.93 and 0.96 respectively, comparable to the rate at which the complete transition matrix at n=12 converges.

In order to fully utilize the available data for determining the value of mutual interactions, as well as identifying the reliability of the estimates, we used an approach that provided a set of values of effective regulatory influences between a pair of gene groups across many different regulatory contexts. Our criterion is more restrictive than the statistical reliability of the average mutual interaction since we are insisting that the regulation be robust in the context of other gene regulatory influences. Thus we are including only the dominant regulatory influences.

The *n*=20, 42, and 72 transition matrices were calculated by randomly selecting 12 of *n* gene groups, and using all 33 observations to determine the mutual influences of these selected 12 groups by a least squares method^1^, yielding 144 parameters out of the total *n*^2^. This process of randomly selecting 12 of the groups and determining their mutual influences was then repeated many times (N) until each of the *n*^2^ influence parameters was estimated at least several times. In the *n*=72 case, we determined the mutual influences of N=600 random *n*=12 sub-networks. Since the fraction of edges estimated in each sampling is p=144/72^2^, the expected number of estimates of each influence was N*p*=16.7, and all influences were estimated at least 4 times, consistent with the binomial distribution result Prob(Binom(N,*p*)<4)= 4.5x10^-5^. This level of sampling and repetition was sufficient to achieve convergence in the 72x72 transition matrix (see Figure 2).

It is possible to statistically estimate transition matrices for *n*>33 because these networks are sparse (most edge influences are small enough to be omitted from consideration). This sparseness can be seen in the exponential distribution of influence magnitudes in Figure 2g of the main text. Out of 72^2^=5184 possible influences, the edge inclusion criterion used in the paper and detailed below identified 337 influences, for an average of 3.7 influences per gene group. The available data, assuming samples are statistically independent, should allow us to identify up to 33x72=2304 influences. This does not include the informational requirement of determining which edges are present and which are absent (by Shannon information theory this is -n^2^(p log(p)+(1-p)log(1-p))=542 bits, where p=337/5184=.065).

**Figure 2** - Convergence in the 72x72 transition matrix as a function of the average number of times each influence was estimated.

When a non-zero-valued edge was included in one of the randomly selected sub-networks, the cumulative effect of all other gene groups on the target gene group of that edge could be approximated as zero on average. This property can be seen by comparing estimates of the value of a particular influence as they are calculated in different sub-networks. These estimates are strongly correlated across all the influences. For example, r=0.43 between vectors composed of influence estimates derived for each edge from independent sub-networks. Over 5184 samples, this correlation is highly significant (p<<0.0001). These estimates are plotted against each other in figure 3 (shown in the right panel is the corresponding plot for edge influences satisfying the edge inclusion criterion below – r=0.91).

**Figure 3** – Scatter plots of the values of regulatory influences as calculated in independent random sub-networks. Left panel is all influences; right panel shows just those influences that pass the edge inclusion criterion described below.

*Edge inclusion*

To analyze network properties such as sparseness, we applied a necessarily arbitrary criterion of including a result only if the mean was greater than two standard deviations. Thus if *a*=abs(mean(*x_i_*))/stdev(*x_i_*)) of the *i* independent estimates of a particular influence was greater than 2, that effective influence was included in the networks. This criterion will exclude edges whose estimated values are not persistent across the regulatory contexts. Numerically this can occur either because the mean is low or the standard deviation is high, corresponding to influences of minimal potency or edges for which we have low confidence in their value, respectively. Analyzing the distributions of the mean values and standard deviations of the estimate repetitions in the included edges versus the excluded edges reveals that edges are excluded primarily because their means are low, rather than because their standard deviations are high (see figure 4). This suggests that noise is not dominating the determination of whether an influence is included in the network or not, and follows from the fact that the standard deviations of included and excluded edges are similarly distributed whereas their means are not.

**Figure 4** – Comparisons of the distributions of the means and standard deviations of the multiple estimates of the edge influence values, in edges passing the inclusion criterion (gray) and those excluded (black).

While the mean to standard deviation ratio threshold, *a*, determines the absolute number of edges we included, varying the choice of this value does not modify the trends we observe by increasing the number of gene groups. As an example, the sparseness of the networks as a function of the edge inclusion threshold and the number of gene groups is shown in figure 5.

**Figure 5** – The sparseness of the influence networks as a function of the number of gene groups has the same qualitative properties regardless of the edge inclusion threshold used. Left panel shows various mean/standard deviation (a) cutoffs.

Our tests of statistical reliability of the regulatory interactions imply that given the same SOM partition, the results can be replicated with a sub-sample of the data and converge reliably to the results we obtained. This implies that random experimental noise does not affect the conclusions. In this analysis and the correlational studies we specifically considered what would be the consequences of performing the same analysis with a different experimental samples, establishing the robustness of the results. Another form of variation is possible through the gene group partitioning.

The choice of SOM algorithm partitioning of genes is not unique. This is however, not a limitation, since any approach, using this or other data, that provides a partitioning of the genes into behaviorally related groups can be used to study the mutual regulatory interactions of the cell. Within the methodology and data we used, the assignment of ontological functions to the gene groups is based upon statistical reliability. The effect of experimental noise on the SOM partitioning would be to reduce the degree to which ontological functions are well separated, spreading assignment more uniformly. The existence of separation is therefore direct evidence that experimental noise does not affect our results, though it is surely possible that it obscured detail from the biological conclusions. Still, it is worth noting that any technique using different data (or other ways of partitioning the same data) that provides distinct ontologically meaningful partitions could augment our results by providing complementary information about cellular regulation.

1. de Bivort, B., Huang, S. & Bar-Yam, Y. Dynamics of cellular level function and regulation derived from murine expression array data. Proc Natl Acad Sci U S A 101, 17687-17692 (2004).
