# Supplementary material for: Empirical Multiscale Networks of Cellular Regulation
Source: PLoS Comput Biol. 2007 Oct 19;3(10):e207. doi: 10.1371/journal.pcbi.0030207 (PMC2041980; doi:10.1371/journal.pcbi.0030207)
Supplement: Text S1 — (80 KB DOC) [file pcbi.0030207.sd002.doc]

# Text S1

Supplementary material for

“Empirical multiscale networks of cellular regulation”

Benjamin de Bivort, Sui Huang and Yaneer Bar-Yam

Harvard University, Harvard Medical School and New England Complex Systems Institute

The mutual regulatory networks we obtained between large groups of genes reveal many details about the specific influences between individual gene groups, as derived from the observed impact of cytokine perturbation. These groups included all 15,000 genes present on the microarrays used to derive this data ^1^, and thus the networks of these groups come close to revealing the large-scale regulatory behavior of the entire cell. While in the main text of this article we primarily explore general properties of the mutual regulatory influences of these gene groups, every entry in the influence matrices constitutes a quantitative prediction of a specific transcriptional regulatory interaction between collections of genes that occurs in the context of cellular perturbation. In this supporting material we first give examples of the kinds of hypotheses generated by our inferred model of cell behavior. Second, we randomly (using computer based random number generation) choose individual hypotheses and consider which of these are intuitive and which are potentially surprising. Lastly we discuss several others that by a more thorough search we found particularly intriguing.

### Types of hypotheses:

The derived influence networks yield many statements of regulatory relationships each of which is a predictive hypothesis. Examples of these kinds of statements include:

1. “Gene group **A** (with functions **GO_A_**) activates/inhibits/doesn’t regulate gene group **B** (with functions **GO_B_**) over **T** hours (with strength **X_AB_**)”
2. “Gene group **A** is primarily an activator/inhibitor/non-regulatory of all other gene groups over **T** hours.”
3. “Among all gene group **A**’s regulatory targets, Gene Group **B** is most strongly activated/inhibited.”
4. “Our estimated value of the interaction between Gene Group **A** and **B** is **X****_AB_** +/- **σ_AB_**, where the interval is at the 95% confidence level.**”**

Each of these categories of statement can be applied to all the scales analyzed, yielding 44,952, 876, 1,752, and 44,952 statements respectively.

We could choose to discuss only subjectively interesting examples of these kinds of statements, however, out of so many possible statements, random chance alone could generate plausible biological predictions. Alternatively, the intuitiveness of the model’s predictions can be explored by considering random predictions. The following examples of type 1 statements at the *n*=20 scale were generated by computationally generating exactly 10 random values each of **A**, **B**, and **T**. They demonstrate that a significant number of the predictions made by our analysis are biologically intuitive:

- Gene group 7 (catabolism/lipid metabolism) inhibits gene group 15 (nucleosome assembly/cell growth/Ras protein signal transduction) over 3.5 hours. It is appropriate that an increase in expression of catabolism-specific genes would associate with a subsequent reduction in the expression of cell-growth specific genes. Cell growth is dependent on the availability of membrane lipid components, and is therefore subject to inhibition by catabolism promoting genes.
- Gene group 18 (lipid catabolism/membrane lipid metabolism/proteolysis and peptidolysis/glutathione conjugation reaction) is weakly self-activating over 1.5 hours. Strong auto-activation is a tell-tale property of bi-stable genetic switches. That this group is not strongly auto-activating over this short interval suggests that it may not be regulated to achieve expression in an all-or-nothing fashion.
- Gene group 14 (base-excision repair/DNA integration) inhibits gene group 15 (nucleosome assembly/cell growth/Ras protein signal transduction) over 3.5 hours. Because DNA damage repair via base-excision repair contributes to a cell-cycle checkpoint before S-phase ^2^, it is appropriate that increased expression of DNA-repair genes would inhibit the expression of nucleosome synthesis genes which are associated with S-phase.
- Gene group 19 (cell-cell adhesion/UDP-N-acetylglucosamine biosynthesis/ transmembrane receptor protein tyrosine phosphatase signaling pathway) inhibits gene group 7 (catabolism/lipid metabolism) over 0.5 hours. This regulation is suggestive of a relationship in which cell-cell adhesion inhibits the catabolic depletion of membrane components.
- Gene group 0 (weakly associated with tRNA synthesis) inhibits gene group 18 (lipid catabolism/membrane lipid metabolism/proteolysis and peptidolysis/ glutathione conjugation reaction) over 3.5 hours. Gene group 0 is not strongly associated with any gene ontology categories, and only weakly associated with tRNA synthesis (See Supporting Table 4). The moderate strength of this interaction may reflect the action of a collection of un-annotated genes or a collection of effector genes too small to be statistically identified.
- Gene group 14 (base-excision repair/DNA integration) weakly inhibits gene group 2 (glycolysis/aerobic respiration/apoptosis/hydrogen transport/ protein folding/glucose metabolism/aldehyde metabolism/translation/proteolysis) over 3 hours. Increases of respiration are associated with the G1 and G2 (growth) cell-cycle phases ^3^. DNA base-excision repair genes are expressed during G1, 3 hours after which corresponds to the end of G1 or the beginning of S in eukaryotic cells. Consistently, G1-specific gene group 14 brings about a reduction in the rate of expression respiration genes during S-phase, when their production rate is known to be lower.
- Gene group 2 (glycolysis/aerobic respiration/apoptosis/hydrogen transport/ protein folding/glucose metabolism/aldehyde metabolism/translation/ proteolysis) activates gene group 19 (cell-cell adhesion/UDP-N-acetylglucosamine biosynthesis/ transmembrane receptor protein tyrosine phosphatase signaling pathway) over 3 hours. This relationship may reflect a connection between oxidative stress and cell surface adhesion which has been proposed as a cellular-level analog of the fight or flight response ^4, 5^.
- Gene group 18 (lipid catabolism/membrane lipid metabolism/proteolysis and peptidolysis/glutathione conjugation reaction) inhibits gene group 16 (oxygen transport) over 1.5 hours. Group 18 is inhibitory of group 16 over all intervals between 0.5h and 2h, but activating over 3 and 3.5h (See Supporting Table 5). This is consistent with the expectation that up-regulation of lipid and protein catabolism genes generates the metabolic substrates of respiration, while simultaneously encouraging the increased uptake of oxygen. The oxygen can be then used in aerobic respiration.
- Gene group 16 (oxygen transport) weakly inhibits gene group 3 (Aerobic Respiration/Cell-cycle/exocytosis/mRNA synthesis/nucleotide metabolism/ translation/transcription) over 3 hours. While it is potentially counter-intuitive that oxygen transport would inhibit aerobic respiration; perhaps this regulatory influence reflects negative-feedback control of the aerobic respiration system as a whole.
- Gene group 17 (oxygen transport/peroxidase reaction) inhibits gene group 13 (phospholipids catabolism/response to stress) over 3.5 hours. Given the inhibitor relationship that lipid catabolism related group 18 has on oxygen transport group 16 over 1.5 hours, this reciprocal inhibition is particularly intriguing. Group 17’s influence on group 13 is activating over 0.5, 1, and 1.5h, but inhibitory over 2, 3, and 3.5h, This stands in almost exact opposition to the regulatory effect of group 18 (lipid catabolism) on group 16 (oxygen). Because these related trends are found in independent pairs of oxygen and lipid catabolism specific groups, it is suggestive of a wider principle in which the oxygen transport and lipid catabolism specific gene groups have opposite reciprocal influences, regardless of which directed influence is activating. The dynamics of a gene that activates its own inhibitor can be characterized as limiting the duration of the expression of that gene, implying that lipid catabolism and oxygen transport mutually limit the expression duration of the other.

While the previous examples were chosen at random, we also identified the following regulatory relationships that are biologically noteworthy out of model predictions obtained from a broader scan of individual influences.

- At *n*=20, group 9 is enriched for mitotic function and is auto-repressing over 3 hours. This regulation may contribute to a limitation on the duration of mitosis-specific gene expression, after induction. Pulse-like expression of mitosis specific genes may reflect their periodic regulation through the cell-cycle.
- At *n*=20, oxygen related gene groups (i.e. 16) have anti-correlated regulatory outputs with group 7 (catabolism / lipid metabolism). This extends the prediction described above in which oxygen transport and lipid catabolism have opposite mutual influence effects, predicting that oxygen transport and lipid catabolism groups have opposite influence effects on all their respective targets.
- At both *n*=12 and 20, gene groups enriched for aerobic respiration, intracellular transport, mRNA synthesis, translation and transcription (groups 0 and 3 respectively) activate all other gene groups except for one: group 3 (cell-type specific/small molecule transport) and group 19 (cell-to-cell adhesion/small molecule transport) at *n*=12 and 20 respectively. At all scales analyzed respiration-translation-transcription gene groups are ubiquitously activating with the exception of small-molecule transport related groups. This suggests that this regulatory relationship is fundamental to all high-levels of regulation in the cell.
- At *n*=20, gene group 3 has a repressing-activating-repressing temporal profile. This temporal pattern may underlie its role in establishing periodic expression of the cell-cycle genes within it.
- At *n*=20, gene group 12 is enriched only for transcription-related genes, and generally does not regulate other groups except over the 2 hour transition, where it is activating. This likely reflects the kinetics of transcript production. Specific experimental evidence ^6^ supporting this prediction includes the observation that lipopolysaccharide (LPS) stimulation induces transcription of tumor necrosis factor alpha, interleukin-6, and interleukin-1β most strongly at 2 hours followed by a decline in transcription in all but the latter. LPS is one of the 33 ligands inducing the transcriptional perturbations in the AfCS data we used to infer the group regulation influences ^1^. This provides support for the hypothesis of 2-hour transcriptional kinetics if LPS up-regulates group 12 very quickly after it is added to the cells. Indeed, group 12 is up-regulated threefold over the 0h to 0.5h interval following the addition of LPS.
- At *n*=20, gene group 4 (mRNA processing) is non-regulatory over all time-steps. As mRNA processing is uniformly required for all translated transcripts, it may be unlikely that genes involved in this function will be involved in regulating transcription of other genes.

References

1. Gilman, A. G. et al. Overview of the Alliance for Cellular Signaling. Nature 420, 703-6 (2002).

2. Offer, H. et al. p53 modulates base excision repair activity in a cell cycle-specific manner after genotoxic stress. Cancer Res 61, 88-96 (2001).

3. Poole, R. K. & Lloyd, D. Changes in respiratory activities during the cell-cycle of the fission yeast Schizosaccharomyces pompe 972h--growing in the presence of glycerol. Biochem J 144, 141-8 (1974).

4. Goligorsky, M. S. The concept of cellular "fight-or-flight" reaction to stress. Am J Physiol Renal Physiol 280, F551-61 (2001).

5. Nachin, L., Nannmark, U. & Nystrom, T. Differential roles of the universal stress proteins of Escherichia coli in oxidative stress resistance, adhesion, and motility. J Bacteriol 187, 6265-72 (2005).

6. Zhou, H. R., Islam, Z. & Pestka, J. J. Kinetics of lipopolysaccharide-induced transcription factor activation/inactivation and relation to proinflammatory gene expression in the murine spleen. Toxicol Appl Pharmacol 187, 147-61 (2003).
